# Tuberculosis of the Scapula, Humerus, and Postero-Lateral Thoracic Wall Without Pulmonary Involvement Mimicking Ewing’s Sarcoma: A Report of a Rare Case

**DOI:** 10.7759/cureus.59131

**Published:** 2024-04-27

**Authors:** Sankalp Yadav

**Affiliations:** 1 Medicine, Shri Madan Lal Khurana Chest Clinic, New Delhi, IND

**Keywords:** cartridge based nucleic acid amplification test (cbnaat), mycobacterium tuberculosis (mtb), scapula, postero-lateral thoracic wall, proximal humerus, joint tuberculosis, chronic shoulder pain

## Abstract

Tuberculosis can present in various atypical forms. The clinical manifestations could involve sites like bones. Tuberculosis of the scapula, humerus, and posterolateral thoracic wall is extremely rare, and diagnosis is challenging due to similarities with conditions like tumors. A young boy presented with swelling of the right shoulder joint for three months. The diagnosis was challenging due to similarities with Ewing’s sarcoma on the initial radiometric workup. A clinical assessment and diagnostic workup with biopsy, a cartridge-based nucleic acid amplification test, and an advanced radiometric investigation resulted in a final diagnosis. He was started on antituberculous drugs for 12 months.

## Introduction

Extrapulmonary tuberculosis is a relatively rare form of tuberculosis [1]. It comprises about 10-15% of all tuberculosis cases [2]. Further, musculoskeletal tuberculosis makes up around 10-35% of all cases of extrapulmonary tuberculosis and only 2% of all tuberculosis cases [3]. It is uncommon to encounter tuberculosis of the flat bones [4]. Less than 1% of all cases of osteoarticular tuberculosis affect the bones of the shoulder joint, and merely a fraction of these involve the scapula [5]. Furthermore, tuberculosis of the proximal humerus and chest wall is very rare. Oftentimes, due to non-specific clinical features, it is misdiagnosed and neglected.

As soft tissues and bone involvement by *Mycobacterium tuberculosis* are rare presentations, in the absence of pulmonary involvement and constitutional symptoms of tuberculosis, the diagnosis can be challenging [6]. Further, it often mimics thoracic wall tumors, tuberculous osteomyelitis, tuberculosis cold abscess, and malignancy [6,7]. Rarely, these lesions may resemble bone tumors or metastatic formations [6].

A rare case of a 13-year-old Indian boy with no pulmonary involvement is reported here. The diagnosis was challenging due to the rarity of the presentation, no pulmonary involvement, and clinical and radiometric investigations mimicking Ewing’s sarcoma.

## Case presentation

A 13-year-old Indian boy born of a non-consanguineous marriage came to the outpatient department as a referral case with complaints of pain and swelling in his right shoulder for three months. The swelling was insidious in onset and without any discharging sinus. There was no fever, night sweats, loss of appetite, or weight loss. Additionally, there was no history of trauma or any major medical or surgical interventions in the past. Also, there was no history of cancer or tuberculosis in his family or contacts. He was a student belonging to a low socioeconomic background living in a small, overcrowded house.

A general examination was suggestive of a young boy with an ectomorphic build. He was hemodynamically stable, with a body mass index of 15.3 kg/m^2^. His systemic examination was unremarkable. The local examination of the right shoulder joint suggested a tender-to-touch, large, swollen joint with a restricted range of movement. Overhead abduction was restricted on the right side. The local skin was erythematous, but there was no raised temperature. The left shoulder joint was normal. Moreover, there was no clubbing, cyanosis, icterus, pedal edema, or lymphadenopathy.

**Figure 1:**
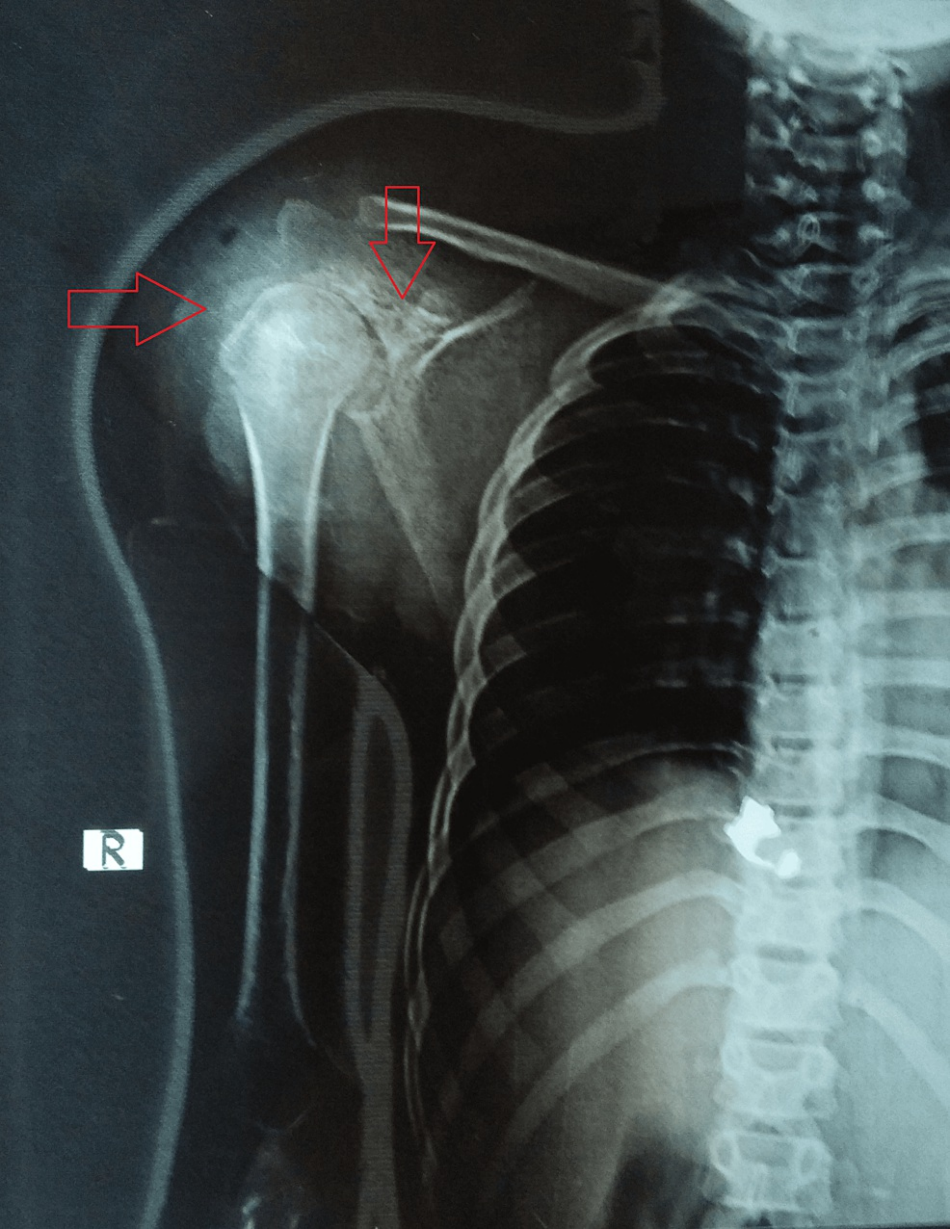
A plain radiograph of the right shoulder suggestive of a soft tissue swelling with erosions of the scapula and osteopenia of the humerus with narrowing of the joint space.

A diagnostic workup was initiated suspecting him to be a case of pyogenic osteomyelitis with differentials including tuberculous osteomyelitis, bone tumors, and fungal osteomyelitis. His laboratory panel was remarkable for a low hemoglobin (6.9 g/dL), a high erythrocyte sedimentation rate (56 mm in the first hour), a high total leukocyte count (12100/cu mm), a raised C-reactive protein (33.0 mg/L), and a positive Mantoux test (20 x 15 mm induration). The rest of the test results, including HIV, hepatitis panel, and induced sputum microscopy, were unremarkable. A radiograph of the right shoulder was suggestive of soft tissue swelling with erosions of the scapula and osteopenia with narrowing of the joint space (Figure 1).

**Figure 2:**
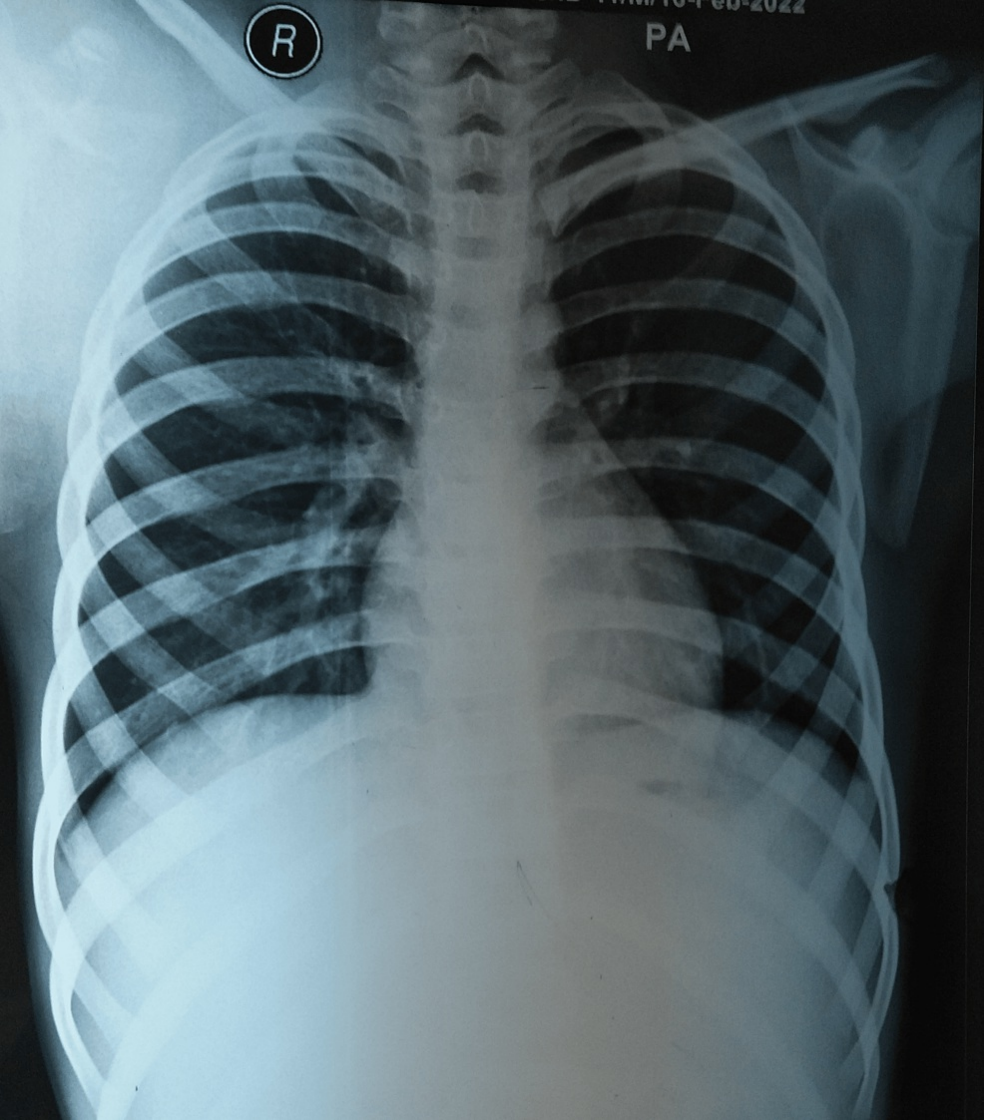
A normal chest radiograph (PA view). PA: posteroanterior

His chest radiograph was normal (Figure 2).

High-resolution sonography of the swelling at the right shoulder was suggestive of a well-defined 9.4 x 6.2 cm heterogenous mass lesion involving the right scapula, right supraspinatus, and infraspinatus muscles. In the Doppler study, there was an increased vascularity with arterial and venous flow and a resistance index of 0.61. A note was made of irregularity or distortion of the cortex of the right acromian process and the coracoid process, suggesting bony erosion of the scapula.

**Figure 3:**
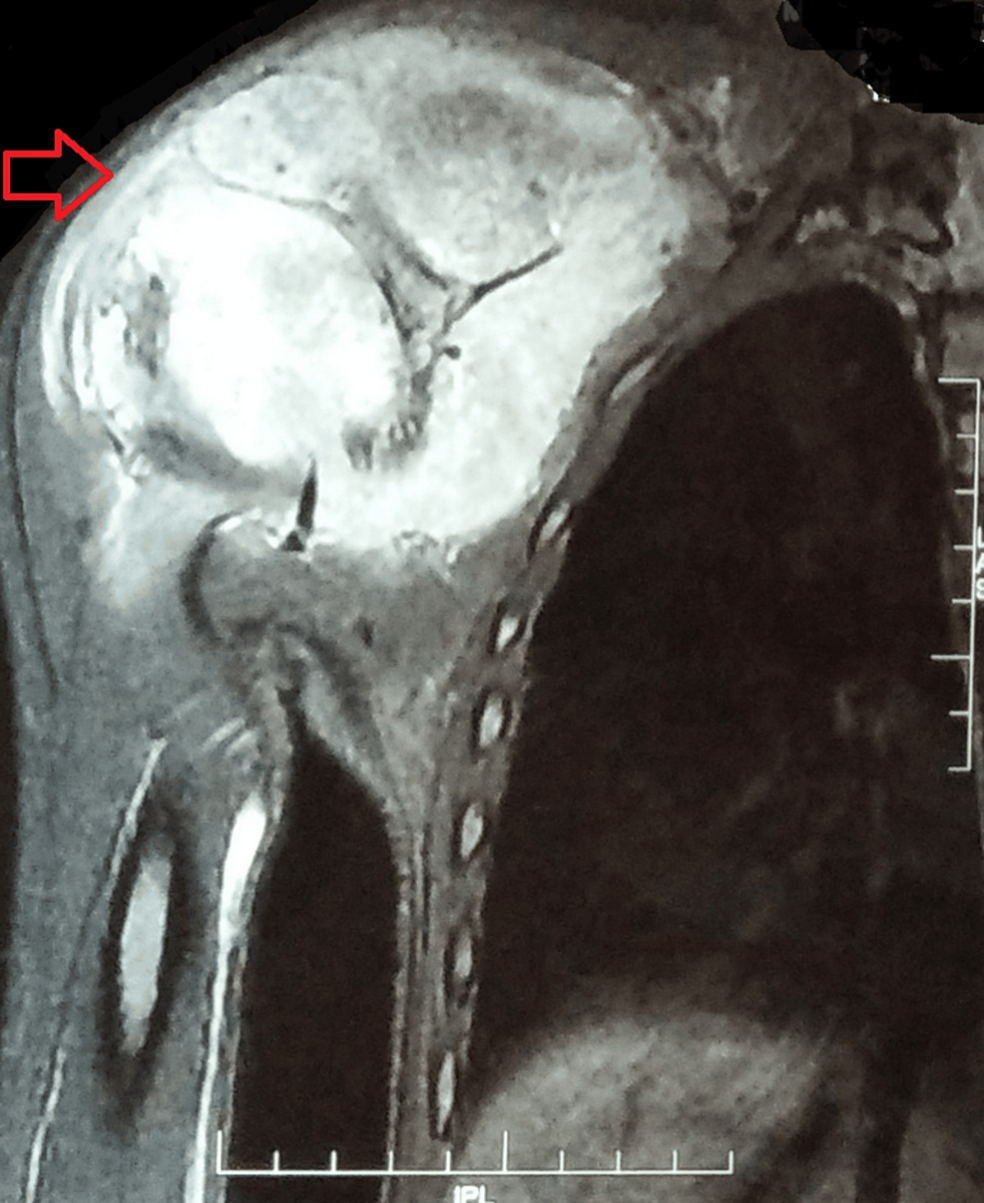
An MRI with contrast suggestive of a lobulated mass lesion on the ventral and dorsal aspects of the right scapula, with evidence of destruction of the body of the scapula. It also shows a focal bony lesion in the right proximal humeral meta-diaphysis MRI: magnetic resonance imaging

**Figure 4:**
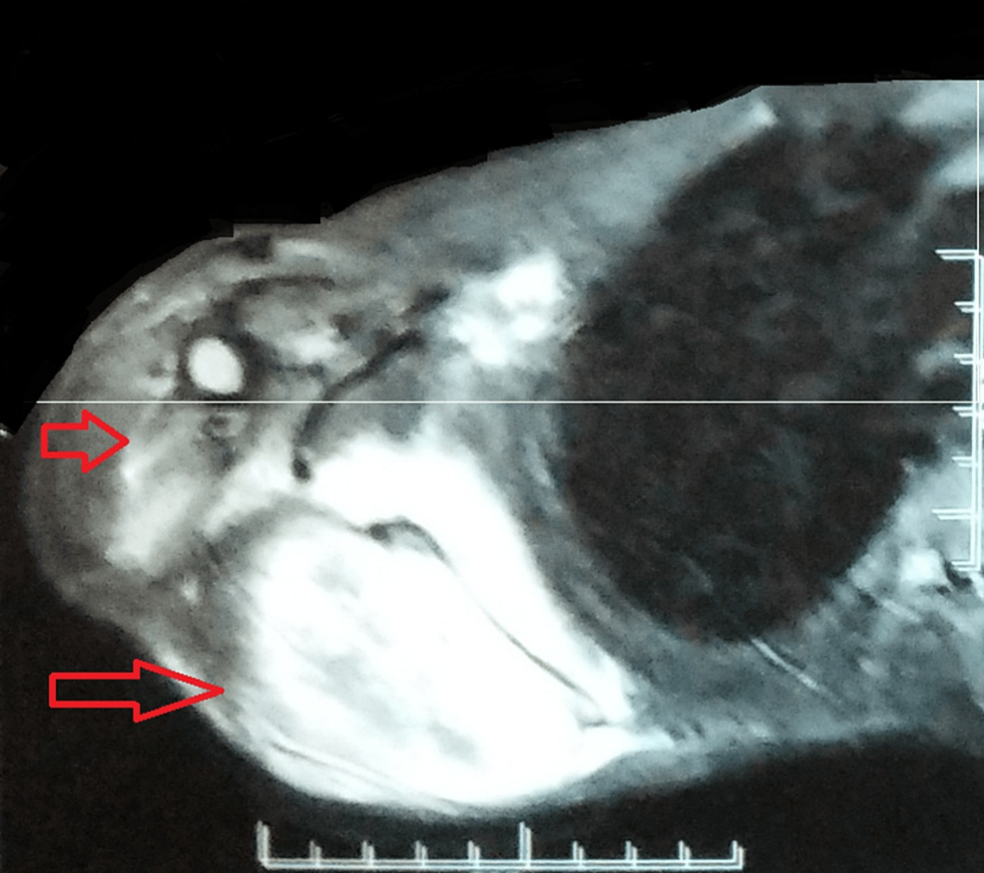
An MRI with contrast suggestive of a lobulated mass lesion on the ventral and dorsal aspects of the right scapula, extending to posterolateral chest wall with evidence of destruction of the body of the scapula MRI: magnetic resonance imaging

Magnetic resonance imaging with contrast was suggestive of a 117 x 82 x 118 cm-sized lobulated mass lesion on the ventral and dorsal aspects of the right scapula with evidence of destruction of the body of the scapula. There was an aggressive periosteal reaction and large soft tissue around the scapula showing restricted diffusion as well as heterogenous post-contrast enhancement. The evidence of a focal bony lesion was also noted in the right proximal humeral meta-diaphysis, measuring 25 x 16 mm. The findings were suggestive of an aggressive neoplasm with an epicenter in the right scapula and a metastatic deposit in the right proximal humerus (Figures 3-4).

**Figure 5:**
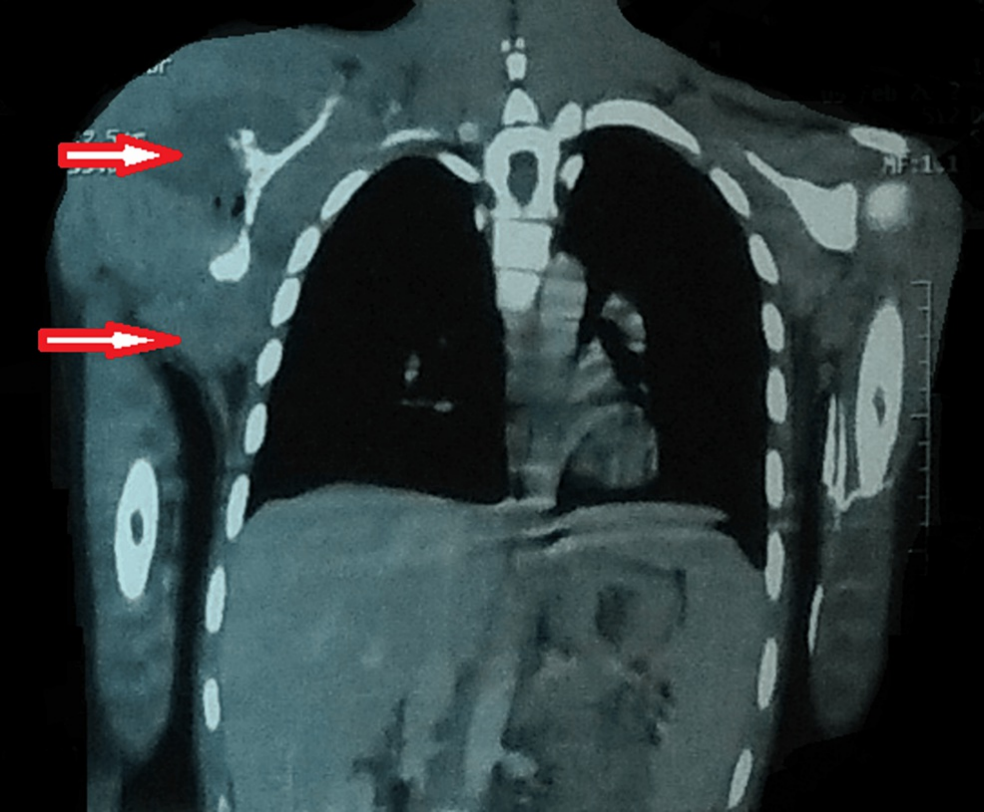
A CT of the chest showing a soft tissue density lesion. Multiple air foci were noted involving the right scapula, with soft tissue extension into the right axillary region involving the subscapular, supraspinatus, and infraspinatus fossa. It showed a soft tissue extension into the glenoid cavity and involved the right posterolateral chest wall CT: computed tomography

A computed tomography of the chest was suggestive of a large, poorly defined, heterogeneously enhancing soft tissue density lesion. Multiple air foci were noted involving the right scapula, with soft tissue extension into the right axillary region involving the subscapular, supraspinatus, and infraspinatus fossa. It showed a soft tissue extension into the glenoid cavity and involved the right posterolateral chest wall (Figure 5).

**Figure 6:**
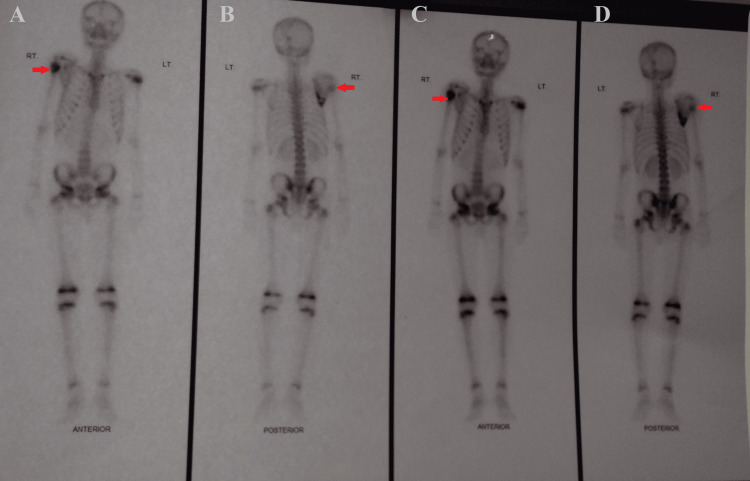
A gamma camera scan is suggestive of a phenotypic lesion with peripherally increased radio-tracer uptake in the proximal end of the right humerus and right scapula. A: radio-tracer uptake in the proximal end of the right humerus; B: radio-tracer uptake in the proximal end of the right humerus and right scapula; C: radio-tracer uptake in the proximal end of the right humerus; D: radio-tracer uptake in the proximal end of the right humerus and right scapula

A gamma camera scan was suggestive of a phenotypic lesion with peripherally increased radio-tracer uptake in the proximal end of the right humerus and right scapula (Figure 6).

A fine-needle aspiration cytology from the right shoulder swelling was suggestive of proliferating pleomorphic cells with enlarged nucleus-to-cytoplasmic in a hemorrhagic background. Inflammatory cells comprising polymorphs and lymphocytes were also noted.

A biopsy was done from the swollen right shoulder at a private center, and the reports of the histopathology were suggestive of tissues with necrotic areas, and a collection of foamy macrophages, neutrophils, lymphocytes, plasma cells, and cellular debris covered by thick fibrous wall. There was no evidence of granuloma or malignancy. Also, histopathology of the right neck lymph node was done, which showed multiple epitheloid cell granulomas with central caseous necrosis and dispersed Langhan’s giant cells consistent with tuberculosis. A cartridge-based nucleic acid amplification test detected *M. tuberculosis *(very low) with no resistance to rifampicin. Additionally, the culture was sterile after 48 days.

Finally, a diagnosis of tuberculosis of the scapula, proximal humerus, and posterolateral thoracic wall was made, and he was commenced on a two-month intensive phase of antituberculous therapy, followed by a 10-month continuation phase with fixed drug combinations of antituberculous drugs. He was counseled for a regular follow-up in the infectious diseases and orthopedics outpatient department and regular visits to physiotherapy clinics. Moreover, he was given a piece of dietary advice and counseling for treatment adherence while maintaining good hygiene and well-ventilated surroundings. To date, patients have completed one year of treatment, with the treatment outcome mentioned in the national data portal as cured at 12 months. However, efforts to assess the clinical improvement at 12 months in the outpatient department proved futile, as he was lost to follow-up.

## Discussion

In countries like India, tuberculosis is a major health issue. The disease is rampant due to unhygienic living conditions, poorly ventilated houses, overcrowding, smoking, and other substance abuse, and a lack of regulatory efforts to prevent an infectious source or person from spreading the disease (although laws are in place, implementing these laws to detain a spreader of tuberculosis is lacking) [8]. The disease manifests in various forms and at various sites [6]. Pulmonary involvement is the commonest, but infection at sites like the scapula, chest wall, and humerus has been reported [9-11].

A total of 1-5% of tuberculosis cases are in the skeletal system [2]. The metaphyses of long bones, including the femur, tibia, fibula, and humerus as well as the vertebrae, pelvis, and skull bones, are the most often impacted bones in children. Even though the infection is brought on by the bacteria’s lymphohematogenous dissemination from the lungs, a primary pulmonary lesion may not be detectable at the time of diagnosis [6]. Further, direct inoculation by a penetrating injury could also result in primary bone and soft tissue involvement [12].

Early in a child's life, radiological and clinical findings were not often clear, making it easy to overlook the diagnosis [6]. The most typical complaints were those of pain and swelling. Seldom-seen symptoms include fever, weight loss, sweats at night, weakness, and pathological fractures [13]. The present case had pain and swelling.

Diagnosis is challenging due to the ambiguity of clinical presentations, early radiographs, and a lack of awareness among primary care physicians. Advanced diagnostic modalities like computed tomography and magnetic resonance imaging are important to determine the extent of bone and soft tissue involvement [6]. However, unless a definitive diagnosis is established by the isolation of *M. tuberculosis* or histopathological findings, one should not rely completely on all these advanced radiometric investigations, including gamma scans, which suggest a preliminary diagnosis of Ewing’s sarcoma [6].

As tuberculosis of the scapula, humerus, and posterolateral chest wall is a paucibacillary condition, the diagnosis could be supported by the histopathological findings of granulomatous inflammation, as seen in the present case. The culture of the specimens for *M. tuberculosis *is a time-consuming procedure and oftentimes results in a delay in diagnosis [6]. Hence, a rapid test like the cartridge-based nucleic acid amplification test should be done upfront [14]. Tuberculin skin test, erythrocyte sedimentation rate, complete blood count, and chest X-ray are auxiliary diagnostic methods [6].

The management is essentially based on antituberculous chemotherapy. Indian guidelines recommend a 12-month antituberculous treatment with the possibility of further extension depending on the clinical status at one year [15]. Surgical interventions should be considered in advanced stages with no clinical improvement or refractory cases, or to remove the sequestrum [16,17]. Additionally, physiotherapy and counseling for treatment adherence are imperative. Regular follow-ups and monitoring are essential, as cases of drug-resistant tuberculosis of the scapula and other bones are also documented in the literature [14].

Previously, cases of Ewing’s sarcoma mimicking tuberculosis and vice versa, have been reported in the literature, but a case of a young boy with concomitant tuberculosis of the scapula, humerus, and posterolateral chest wall mimicking Ewing’s sarcoma has never been reported to the best of my knowledge [6].

## Conclusions

This case stresses the need for a detailed clinical assessment with an open mind for various atypical presentations of a common disease. A timely diagnostic workup led to the definitive diagnosis in this case, resulting in avoiding superfluous management for Ewing’s sarcoma. It is also essential that cases similar to this are reported on a large scale, especially from endemic countries, as this will help in making or modifying a specific management protocol for the best outcomes.
